# Effect of decaffeinated coffee on function and nucleotide metabolism in kidney

**DOI:** 10.1007/s11010-017-3131-9

**Published:** 2017-08-02

**Authors:** I. M. Rybakowska, R. Milczarek, E. M. Slominska, R. T. Smolenski

**Affiliations:** 1 Department of Biochemistry and Clinical Physiology, Medical University of Gdansk, Debinki 1, 80-211, Gdansk, Poland; 2 Department of Pharmaceutical Biochemistry, Medical University of Gdansk, Gdansk, Poland; 3 Department of Biochemistry, Medical University of Gdansk, Gdansk, Poland

**Keywords:** Decaffeinated coffee, Ecto5′-nucleotidase, Adenosine, Creatinine

## Abstract

Little is known about the effects of coffee that are not related to the presence of caffeine. The aim of the study was to analyse changes in kidney function and nucleotide metabolism related to high intake of decaffeinated coffee. Mice consumed decaffeinated coffee extract for two weeks. Activities of AMP deaminase, ecto5′-nucleotidase, adenosine deaminase, purine nucleoside phosphorylase were measured in kidney cortex and medulla by analysis of conversion of substrates into products using HPLC. Concentration of nucleotides in kidney cortex, kidney medulla and serum were estimated by HPLC. Activity of ecto5′-nucleotidase increased from 0.032 ± 0.006 to 0.049 ± 0.014 nmol/mg tissue/min in kidney cortex of mice administered high-dose decaffeinated coffee (HDC) together with increase in cortex adenosine concentration and decrease in plasma creatinine concentration. HDC leads to increased activity of ecto5′-nucleotidase in kidney cortex that translates to increase in concentration of adenosine. Surprisingly this caused improved kidney excretion function.

## Introduction

It is estimated that more than 59% of adults in the United States drink coffee beverages and similarly in other developed countries [1]. While consumption of coffee is prevalent, approximately 15% of the U.S. population has stopped drinking coffee altogether, citing concerns about health, coffee reduction has been a strategy to prevent urinary tract symptoms such as bladder pain syndrome [1]. There are reports of randomized controlled trials on chronic consumption of coffee that confirmed a small but statistically significant increase in systolic blood pressure and diastolic blood pressure [2].

Although caffeine consumption in the 200–300 mg range increases urinary calcium levels in both younger and older individuals, the predominance of data suggests that caffeine has a greater impact on calcium metabolism and bone in older people [3]. While caffeine intake may increase urine calcium excretion, caffeine-containing beverages have been associated with a lower risk of nephrolithiasis. Caffeine intake is associated with a lower risk of incident kidney stones [4].

The effects of coffee on the metabolism and genotoxicity of the dietary carcinogen were also investigated. Coffee increased the expression of CYP1A2 by 16-fold in the 5% coffee-treated group, and approximately half of this inductive effect was attributed to caffeine. Coffee also increased the expression of enzymes involved in the detoxication of dietary carcinogens such as glutathione S-transferase alpha [5]. There is some evidence that caffeine slightly increases the risk of cancer at the major organs [6] but there are also findings showing that coffee may exert protective effect against colorectal cancer [7–9]. There is also some evidence that coffee results in an increase in some markers of inflammation.

Decaffeinated coffee intake was associated with a small and clinically irrelevant decrease in mean diastolic blood pressure [10]. Coffee and decaffeinated coffee have the ability to improve performance during a resistance exercise protocol [11]. Other compounds instead of caffeine including polyphenols or other may be beneficial or harmful [12]. Because of the occurrence of numerous naturally occurring polyphenolic antioxidants in coffee and tea, these beverages have attracted attention as a possible safe means to protect against reactive oxygen species [13]. A number of foods and beverages contain isothiocyanates, polyphenols, diterpenes and other components that have been reported to induce glutathione S-transferases, enzymes that detoxify a wide range of dietary and environmental contaminants [14–16]. The effects of the active compounds in decaffeinated coffee on kidney function must be considered.

Nucleotides build DNA, RNA and other important molecules like coenzymes, ATP with high energetic bonds. The physiological role of the reaction catalyzed by enzymes nucleotide metabolism AMP deaminase (AMPD) and ecto5′-nucleotidase (e5NT), adenosine deaminase (ADA) in kidney is mainly the regulation of adenine nucleotide pools in the cell and control of adenosine concentration. It is well proven that caffeine can affect adenosine receptors as its receptor antagonist and it also inhibits phosphodiesterase responsible for transformation of cAMP to AMP. cAMP among others regulates triacylglycerol hormone-sensitive lipase in adipose tissue [17, 18]. The evidence suggests that adenosine receptors inhibit lipolysis by decreasing the activity of adenylate cyclase, which makes adenosine receptors a potential target in obesity and diabetes [17]. Adenosine receptors are located in the whole body, but mainly they can be found in brain, heart, blood vessels, kidneys and adipose tissue. In contrast to coffee effect related to caffeine, little is known about the effect of compounds other than caffeine present in coffee.

The aim of this study was to evaluate the effect of decaffeinated coffee on enzymes of nucleotide conversions, adenosine level and energetic equilibrium in kidneys and its relation to basic aspects of kidney function.

## Materials and methods

### Coffee preparation

Coffee espresso was prepared from 7 g decaffeinated coffee powder in 25 ml of water by high-pressure coffee machine. 0.33 ml of caffeine coffee and 0.33 ml decaffeinated coffee were diluted to 5 ml of water for daily dose, respectively, of Caffeine (Caff) and low-dose decaffeinated coffee (LDC) for mice. 3.3 ml of decaffeinated coffee extract was diluted to 5 ml of water which was the approximate daily high dose for mice—HDC.

### Animals

The study was approved by Medical University of Gdansk Ethics Committee for the Animal Experiments—29/2012. Three-month-old mice C57/BL-6 were treated for two weeks without limitation (about 5 ml/day/mouse) with water or solution of caffeine coffee (Caff), low-dose decaffeinated coffee (LDC) and high-dose decaffeinated coffee (HDC) extract. Coffee extract contained from 0.68 to 6.8 mg dissolved coffee per ml, respectively, for Caff, LDC and for HDC. After this time mice were anaesthetized with 2.5 µg xylazine and 43 µg ketamine/g weight of mice and were organs collected. The blood was collected from caval vein. For nucleotide determination, one kidney was frozen with tongs precooled in liquid nitrogen, and for activity determination, the kidney was divided into cortex and medulla and then frozen.

### Measurement of nucleotide concentrations

Kidneys were lyophilisated, separated into cortex and medulla and then extracted with 25-fold volume of in 0.4 M HClO_4_. After that the samples were centrifuged for 5 min at 14,000 rpm in 4 °C. The supernatant was neutralized with 2 M KOH and then incubated for 10 min in ice. The supernatant obtained after 5 min centrifugation at 14000 rpm in 4 °C was analysed by HPLC. Serum was extracted in the same volume of 1.3 M HClO_4_, then centrifuged and supernatant neutralized with 3 M K_3_PO_4_. After centrifugation, supernatants were analysed by HPLC.

### Measurement of nucleotide metabolism enzyme activities

The activities of AMP deaminase (AMPD), ecto5-nucleotidase (e5NT), adenosine deaminase (ADA), purine nucleoside phosphorylase (PNP) were measured by determination of reaction products with HPLC as described before [19].

Kidneys were homogenized at 4 °C in 9 volume of buffer (150 mM KCl, 20 mM Tris, 1 mM EDTA, 1 mM dithiothreitol, pH 7.0). A portion of crude homogenate was taken for determination of 5NT after 1 h in 4 °C. Remaining homogenate was centrifuged at 3400 rpm at 4 °C for 10 min and then added in volume 50 µl to the specific for particular enzyme buffer and incubated with 50 µl of suitable substrate for enzyme buffer at 37 °C. Buffer for determination of AMPD consisted of 20 mM TRIS, 150 mM KCl, 1 mM dithiothreitol, for determination 5NT consisted of 50 mM TRIS, 5 mM MgCl_2_, 10 mM β glycerolphosphate, 0.1% TRITON X-100. Buffer for determination of ADA consisted of 50 mM TRIS, and for PNP consisted of 50 mM NaH_2_PO_4_. The substrate concentrations were 25 mM AMP for AMPD, 1 mM adenosine for ADA, 1 mM inosine for PNP and 0.2 mM AMP for 5NT. The reaction was terminated by adding 100 µl 1.3 M HClO_4_ and after centrifugation neutralized to pH 6–7 with 3 M K_3_PO_4_. Centrifuged extracts were analysed by HPLC [20].

### Statistical analysis

Data are expressed as mean ± standard deviation (SD). Values were compared with the One-Way Anova of Variance (ANOVA). Tukey’s post hoc test was used for further determination of the significance of differences which were considered significant at *p* < 0.05.

## Results

**Fig. 1:**
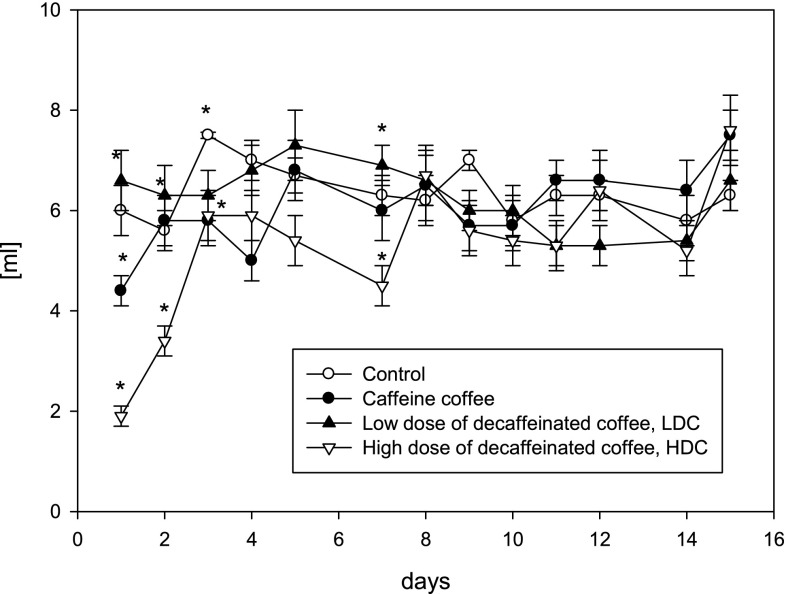
The volume of fluid ingested in C (water), Caffeine coffee, low-dose decaffeinated coffee LDH or HDC high-dose decaffeinated coffee groups. Values are mean ± SD, *n* = 6, *p < 0.05

**Fig. 2:**
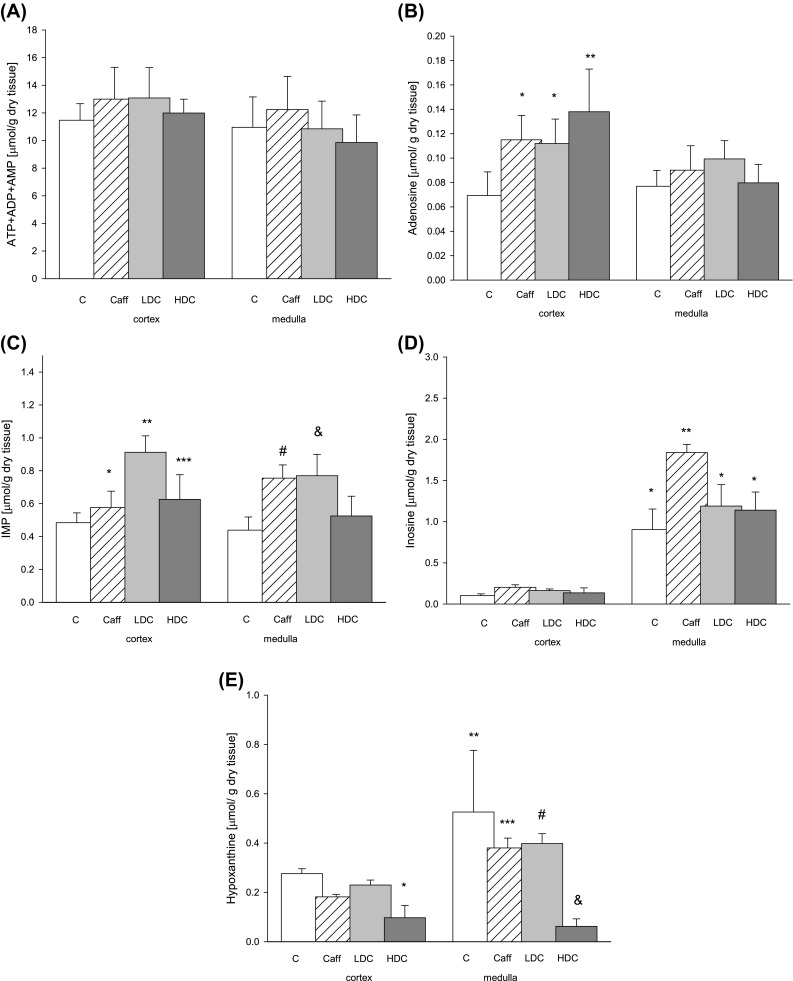
Concentrations of **a** adenine nucleotides **b** adenosine **c** IMP **d** inosine **e** hypoxanthine in kidneys snap-frozen in liquid nitrogen and after lyophilisation divided into cortex and medulla; *C* control mice that drank water, *Caff* mice drinking caffeine coffee, *LDC* mice drinking low-dose decaffeinated coffee, *HDC* mice drinking high-dose decaffeinated coffee. Values are mean ± SD, *n* = 6, b *p < 0.05 versus C cortex; **p < 0.05 versus C cortex and all medulla c *p < 0.05 versus LDC cortex and LDC medulla; **p < 0.05 versus all cortex and C, Caff, HDC medulla; ***p < 0.05 versus C medulla; ^#^ *p* < 0.05 versus C cortex and C, HDC medulla and * p* < 0.05 versus LDC medulla d *p < 0.05 versus all cortex; **p < 0.05 versus all cortex and LDC, HDC medulla e *p < 0.05 versus C cortex; **p < 0.05 versus all cortex and HDC medulla; ***p < 0.05 versus HDC medulla; ^#^ *p* < 0.05 versus Caff, HDC cortex and HDC medulla and* p* < 0.05 versus C cortex

Mice that received decaffeinated coffee ingested similar volume to control mice except first three days (Fig. 1). Concentration of adenine nucleotide pool did not change between groups in kidney cortex and medulla (Fig. 2a). Concentration of adenosine (Ado) increased twofold in kidney cortex mice drinking high-dose decaffeinated coffee (HDC) and increased also but it was less in Caff and LDC group. Such changes in adenosine concentration were not observed in kidney medulla (Fig. 2b). Moreover concentration of IMP and inosine did not change in HDC group but increased in Caff group in medulla (Fig. 2c, d). We noted increase in inosine concentration in Caff and LDC group in cortex and twofold increase in Caff group in medulla (Fig. 2d).

**Fig. 3:**
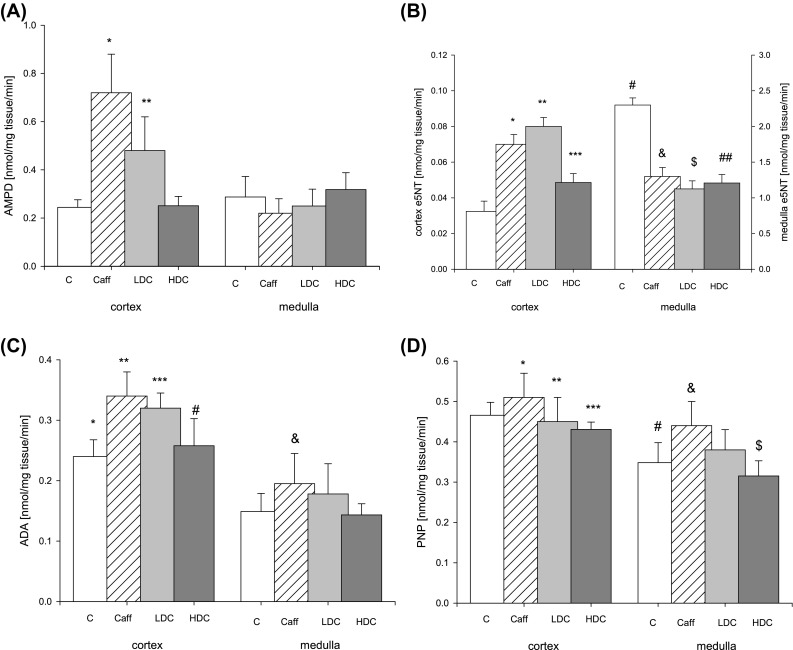
Activity of **a** AMP deaminase (AMPD), **b** ecto5′-nucleotidase (e5NT), **c** adenosine deaminase (ADA), **d** purine nucleoside phosphorylase (PNP) in kidney divided into cortex and medulla and then frozen. *C* control mice that drunk water, *Caff* mice drunk caffeine coffee, *LDC* mice treated low-dose decaffeinated coffee, *HDC* mice treated high-dose decaffeinated coffee. Values are mean ± SD *n* = 6, **a** *p < 0.05 versus all cortex and all medulla; **p < 0.05 versus all cortex and C, Caff, LDC medulla **b** *p < 0.05 versus C, HDC cortex and all medulla; **p < 0.05 versus C, HDC cortex and all medulla; ***p < 0.05 versus all cortex and C medulla; ^#^ *p* < 0.05 versus all cortex and all medulla; and* p* < 0.05 versus Caff, LDC cortex and all medulla; ^$^ *p* < 0.05 versus HDC medulla; ^##^ *p* < 0.05 versus C cortex c *p < 0.05 versus Caff, LDC cortex and C, HDC medulla; **p < 0.05 versus HDC cortex and all medulla; ***p < 0.05 versus all medulla; ^#^ *p* < 0.05 versus LDC, HDC medulla; and* p* < 0.05 versus C, HDC medulla **d** *p < 0.05 versus C, LDC, HDC medulla; **p < 0.05 versus C, HDC medulla; ***p < 0.05 versus HDC medulla; ^#^ *p* < 0.05 versus C cortex; and *p* < 0.05 versus all medulla; ^$^ *p* < 0.05 versus C cortex

Mice kidney activities of AMP deaminase (AMPD) did not change in HDC group in cortex and in all groups in medulla, whereas it increased about twofold in Caff and LDC group (Fig. 3a). We noticed increase of ecto 5′-nucleotidase (e5NT) in kidney cortex of HDC mice and greater increase in Caff and LDC group and inverse relationship in kidney medulla: about twofold decrease in kidney medulla of examined mice compared to control mice (Fig. 3b). Activities of adenosine deaminase (ADA) increased in cortex Caff and LDC group (Fig. 3c). We observed also increase in purine nucleoside phosphorylase (PNP) activity in Caff mice in medulla, respectively, to LDC and HDC group (Fig. 3d).

**Fig. 4:**
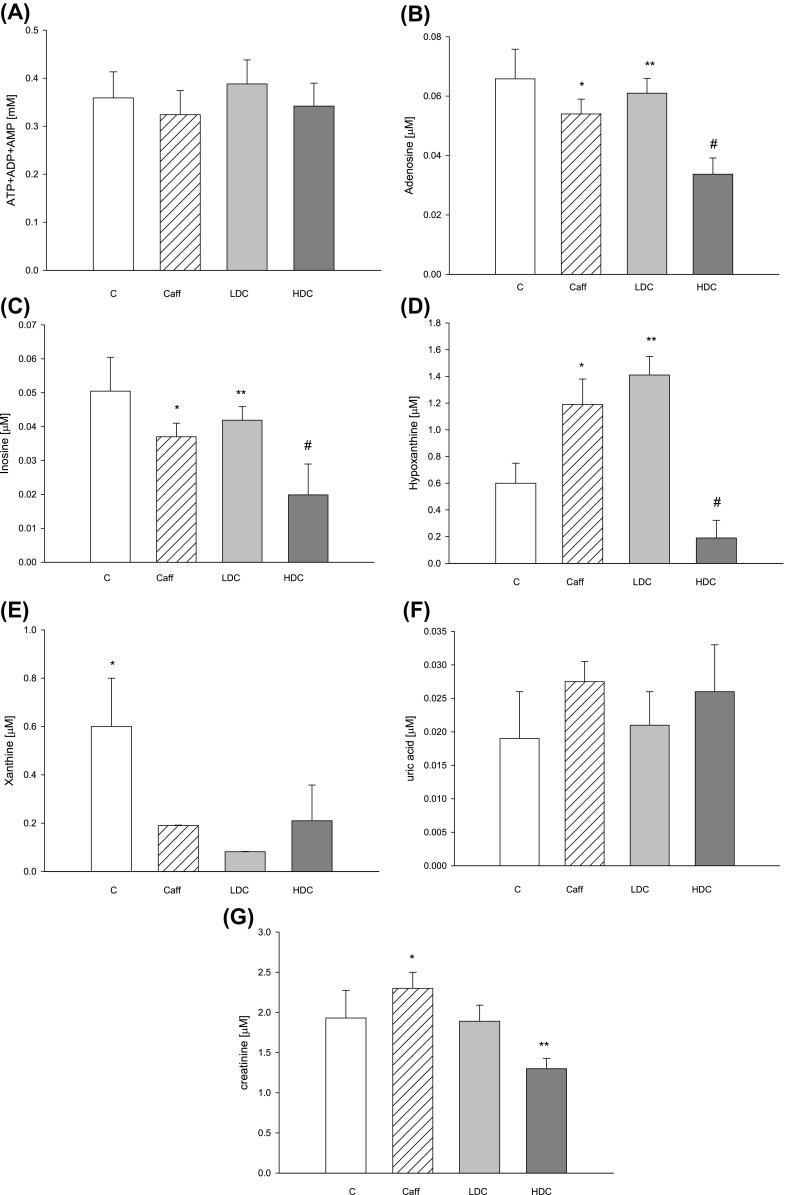
Concentrations of **a** adenine nucleotide, **b** adenosine, **c** inosine, **d** hypoxanthine, **e** xanthine **f** uric acid, **g** creatinine in serum; *C* control mice drunk water, *Caff* mice drunk caffeine coffee, *LDC* mice-treated low-dose decaffeinated coffee, *HDC* mice treated high-dose decaffeinated coffee. Values are mean ± SD, *n* = 6, b *p < 0.05 versus C, HDC; **p < 0.05 versus HDC, ^#^ *p* < 0.05 versus C; **c** *p < 0.05 versus C; **p < 0.05 versus HDC; ^#^ *p* < 0.05 versus C **d** *p < 0.05 versus C, HDC; **p < 0.05 versus C, HDC; ^#^ *p* < 0.05 versus Caff, LDC e *p < 0.05 versus all **g** *p < 0.05 versus LDC, HDC; **p < 0.05 versus C, Caff

Serum concentrations of adenine nucleotide pool and uric acid did not change (Fig. 4a, f). What is interesting we observed twofold decrease in creatinine concentration in serum of HDC mice (Fig. 4g). Moreover we saw decrease in concentrations of adenosine, inosine, hypoxanthine, xanthine in serum of HDC mice (Fig. 4b–e). We saw also higher concentration of adenosine, inosine, hypoxanthine compared to HDC mice in serum (Fig. 4b–d).

## Discussion

Increase in ecto5′-nucleotidase activity in kidney cortex was a major change observed in mice drinking decaffeinated and also caffeine coffee. 5′nucleotidases dephosphorylate non-cyclic nucleoside monophosphates to nucleosides and inorganic phosphate. The presence in the human genome of at least seven genes for 5′-nucleotidases suggests that these enzymes perform important metabolic functions [21]. The existence of common motifs suggests a common catalytic mechanism for all intracellular 5NT. Some 5′-nucleotidases are ubiquitous cN-II, cdN, mdN; others display tissue-specific expression cN-I and cN-III. All 5′nucleotidases have relatively broad substrate specificities. Although e5NT has broad substrate specificity, AMP is considered to be the major physiological substrate. Independent of the enzymatic function, the protein acts as co-receptor in T cell activation and as cell adhesion molecule, e5NT is variably expressed in a wide number of cell types under physiological and pathological conditions. In neuronal cells, e5NT expression is linked to development. The proximal promoter region of the gene contains a number of tissue-specific elements [21]. In our study, pathway that converts AMP to adenosine is activated by increase in activity of e5NT in kidney cortex mice. However, we observed decreased e5NT activity in kidney medulla.

AMP deaminase which catalyzes conversion of AMP to IMP plays important role in regulation of nucleotide metabolism. Physiological role of reaction catalyzed by kidney enzyme stands on keeping correct values of energetic adenylate charge $${{\left( {\left[ {\text{ATP}} \right] + {\raise0.7ex\hbox{$1$} \!\mathord{\left/ {\vphantom {1 2}}\right.\kern-0pt} \!\lower0.7ex\hbox{$2$}}\left[ {\text{ADP}} \right]} \right)} \mathord{\left/ {\vphantom {{\left( {\left[ {\text{ATP}} \right] + {\raise0.7ex\hbox{$1$} \!\mathord{\left/ {\vphantom {1 2}}\right.\kern-0pt} \!\lower0.7ex\hbox{$2$}}\left[ {\text{ADP}} \right]} \right)} {\left( {\left[ {\text{ATP}} \right] + \left[ {\text{ADP}} \right] + \left[ {\text{AMP}} \right]} \right)}}} \right. \kern-0pt} {\left( {\left[ {\text{ATP}} \right] + \left[ {\text{ADP}} \right] + \left[ {\text{AMP}} \right]} \right)}}$$ phosphorylation potential $$\left( {{{\left[ {\text{ATP}} \right]} \mathord{\left/ {\vphantom {{\left[ {\text{ATP}} \right]} {\left( {\left[ {\text{ADP}} \right] \times \left[ {\text{Pi}} \right]} \right)}}} \right. \kern-0pt} {\left( {\left[ {\text{ADP}} \right] \times \left[ {\text{Pi}} \right]} \right)}}} \right)$$ and free energy hydrolysis of ATP [20, 22]. In kidneys, purine nucleotide cycle plays fundamental role in protecting the purine ring against degradation. It is also responsible for generation of ammonia and fumarate, which increases efficiency and relation between glycolysis and Krebs cycle. Moreover, it regulates level of AMP, which is the main source of adenosine in kidneys [23]. Concentrations of adenine nucleotides $$({\text{ATP}} + {\text{ADP}} + {\text{AMP}})$$ did not change in cortex and medulla but changed AMPD activity in Caff and LDC group in cortex in vivo after drinking coffee.

Activity of PNP, enzyme metabolizing inosine to hypoxanthine, decreased slightly in kidney cortex and medulla HDC mice what is reflected in trend for increase in concentration of inosine and decrease of hypoxanthine. However, we noticed trend for increase in activity of PNP in cortex and increase in Caff group with increase in concentration of hypoxanthine compared to HDC group. Increase in activity of PNP in Caff group in kidney was not consistent with changes in concentration of inosine that increase. However, this could be the result of higher activity of ADA.

Earlier study demonstrated that hyperfiltration, which is an early marker of diabetic nephropathy, is connected with greater capacity of kidneys to produce and excrete adenosine [24, 25]. Hyperfiltration is an action of atrial natriuretic factor (ANF) and glucagon. There were studies which employ adenosine deaminase, which converts adenosine to inosine, to eliminate effects of intrarenal adenosine on glomerular hyperfiltration. Results showed that in rats treated with ADA, ANF and glucagon increase glomerular filtration (GFR) dramatically, while treatment only with ADA showed no changes in GFR and renal plasma flow. It is believed that renal endogenous adenosine prevents hyperfiltration which is caused by ANF and glucagon [16]. It is possible that decaffeinated and caffeine coffee causes increase of filtration and production of adenosine (Fig. 2b). Lower concentration of adenosine in Caff and LDC cortex kidney than in HDC may be the result of higher activity of ADA causing increase of inosine in Caff group. Adenosine is rapidly metabolized, so its serum concentrations (Fig. 4b) may not reflect its in vivo concentration in circulating blood.

Adenosine is locally produced in kidney as a by-product of ATP metabolism. It is connected with regulation of renal blood flow and glomerular filtration rate (GFR). It is able to modulate renal vascular function by adenosine (A_1_) receptors [26]. Adenosine causes vasoconstriction of the afferent blood vessel and vasodilatation of the efferent blood vessel. In most vascular beds, adenosine plays role of vasodilator, unlike in kidney. Moreover, it modulates various renal mechanisms, such as tubuloglomerular feedback, tubular transport and renin release. Its vasoactivity with the association of intracellular energetic metabolism and other actions, including inhibition of platelet aggregation, sympathetic neurotransmission, lipolysis and stimulation of glucose transport and oxidation, indicate that physiological role of adenosine is to inhibit energy consumption in some tissues and increase energy supplies in others [25, 27]. During inflammatory condition which happens in obesity and type 2 diabetes, adenosine is released in order to decrease the production of cytokines [28, 29]. It was reported that sensitivity of several tissues to adenosine, including kidney, may increase as a result of insulin-dependent diabetes. Elevated levels of adenosine may bring important physiological consequences, especially in early stage of diabetes [25]. It is possible that increased concentration of adenosine in kidney cortex mice is balanced by lack of change in adenosine kidney medulla concentration. Decaffeinated and caffeine coffee leads to increased activity of ecto5′-nucleotidase in kidney cortex that translates to increase in concentration of adenosine. Surprisingly, this caused improved kidney excretion function.

Results of this study demonstrated that high consumption of decaffeinated coffee increases adenosine formation in kidney cortex in ecto5′-nucleotidase pathway. This was associated with increased creatinine excretion.
